# KPC-luciferase-expressing cells elicit an anti-tumor immune response in a mouse model of pancreatic cancer

**DOI:** 10.1038/s41598-024-64053-0

**Published:** 2024-06-13

**Authors:** Daniele Pereira Ferrari, Fernanda Ramos-Gomes, Frauke Alves, M. Andrea Markus

**Affiliations:** 1 Translational Molecular Imaging, Max-Planck-Institute for Multidisciplinary Sciences, Hermann Rein‑Straße 3, 37075 Göttingen, Germany; 2 Institute of Diagnostic and Interventional Radiology, University Medical Center Göttingen, Robert-Koch-Str. 40, 37075 Göttingen, Germany; 3 Department of Haematology and Medical Oncology, University Medical Center Göttingen, Robert-Koch-Str. 40, 37075 Göttingen, Germany

**Keywords:** Cancer imaging, Tumour immunology

## Abstract

Mouse models for the study of pancreatic ductal adenocarcinoma (PDAC) are well-established and representative of many key features observed in human PDAC. To monitor tumor growth, cancer cells that are implanted in mice are often transfected with reporter genes, such as firefly luciferase (Luc), enabling in vivo optical imaging over time. Since Luc can induce an immune response, we aimed to evaluate whether the expression of Luc could affect the growth of KPC tumors in mice by inducing immunogenicity. Although both cell lines, KPC and Luc transduced KPC (KPC-Luc), had the same proliferation rate, KPC-Luc tumors had significantly smaller sizes or were absent 13 days after orthotopic cell implantation, compared to KPC tumors. This coincided with the loss of bioluminescence signal over the tumor region. Immunophenotyping of blood and spleen from KPC-Luc tumor-bearing mice showed a decreased number of macrophages and CD4^+^ T cells, and an increased accumulation of natural killer (NK) cells in comparison to KPC tumor mice. Higher infiltration of CD8^+^ T cells was found in KPC-Luc tumors than in their controls. Moreover, the immune response against Luc peptide was stronger in splenocytes from mice implanted with KPC-Luc cells compared to those isolated from KPC wild-type mice, indicating increased immunogenicity elicited by the presence of Luc in the PDAC tumor cells. These results must be considered when evaluating the efficacy of anti-cancer therapies including immunotherapies in immunocompetent PDAC or other cancer mouse models that use Luc as a reporter for bioluminescence imaging.

## Introduction

Pancreatic ductal adenocarcinoma (PDAC) is a malignancy with high incidence and mortality, and it is characterized by its aggressive biology and poor prognosis^1^. The current treatments for PDAC are limited, highlighting the importance of finding novel strategies. Murine cancer models play a central role in understanding PDAC progression and pathophysiology, as well as in testing novel treatments^2,3^. As most PDAC patients show mutations in the KRAS and Tp53 genes^4^, the KPC (Kras^G12D/+^; Trp53^R172H/+^; P48-Cre) mouse is the most frequently used genetically engineered model in pancreatic cancer research, due to its reproducibility of the immune microenvironment of human PDAC, which allows the evaluation of preclinical therapeutic agents including immune therapy^5–7^. The tumors that arise spontaneously in the KPC mice have histological similarities to human PDAC, tending to be highly stromal with dense desmoplasia. Still, they can take a long time to form, increasing the costs of the experiment^8^. It is therefore common practice to employ transplantation models using human or mouse PDAC cells isolated from primary tumors that are implanted into recipient mice via subcutaneous, intravenous, or orthotopic injection. In particular, orthotopic models are attractive because the tumors grow in the native organ, and distant metastasis occurs spontaneously and rapidly throughout the abdomen in a manner consistent with clinical human disease^9^. Additionally, orthotopic implantation of PDAC cells recapitulate the tumor microenvironment of human PDAC more accurately than subcutaneous tumor models, is cost-effective, and at the same time reduces the degree of tumor heterogeneity seen in genetically engineered mouse models^7,10^.

In vivo live imaging is a valuable non-invasive tool for investigating cancer progression and unveiling the evasive response patterns of tumors. In preclinical research, cancer cells can be labeled with optically detectable markers to identify the location and putative spread of tumors. This methodology commonly employs bioluminescence generated by reporter proteins such as firefly luciferase (Luc^11,12^). The use of Luc in bioluminescence imaging (BLI) is suitable for quantitative real-time analysis of cancer development at the cellular level in living organisms and enables monitoring of the disease progression^13^. Bioluminescence occurs during the catalytic ATP-dependent reaction of Luc and its substrate d-luciferin, which yields photon emission at 560 nm ^14^, thus providing an imaging method to detect living tumor cells with high sensitivity and specificity (without autofluorescence and limited to the gene-expressing cells) and allowing repetitive measurements due to fast body clearance^15^.

Although BLI is extensively used in the field of cancer imaging and considered an attractive route for monitoring tumor growth and therapeutic responses, some studies have reported a reducing tumorigenesis and metastasis effect in syngeneic host mice where Luc-expressing cells were implanted^16–18^. For example, in a breast cancer model, the growth rate of 4T1Luc-expressing tumors was lower than those from its parental clone 4T1, and fewer metastases were detected in immunocompetent mice implanted with 4T1Luc, although the in vitro proliferation rates of both cell lines were similar^16^. Furthermore, tumor growth retardation was found by the murine colon cancer cells CT26/Luc inoculated in immunocompetent BALB/c mice, but not in immunodeficient Nu/Nu mice^19^. However, the influence of Luc expression in the development of PDAC tumors has not been investigated yet. The expression of foreign proteins, such as Luc in cancer cells can work as an antigen that is targeted by the immune system, thus inducing an adaptive immune response and confounding the validity of preclinical conclusions^19^.

In this study, we show that the expression of Luc in PDAC cells derived from the KPC mouse impacts the development of primary tumors and metastasis, in an orthotopic syngeneic model using immunocompetent mice. The presence of Luc in KPC cells induces a potent immune response, illustrated by high T cell activation and infiltration in the tumor, leading to complete and permanent regression. Thus, researchers might consider these results before choosing an immunocompetent PDAC mouse model suitable for monitoring tumor progression by in vivo bioluminescence imaging in cancer studies.

## Methods

### Cells

The KPC cell line was provided by Prof. Volker Ellenrieder (Clinic for Gastroenterology, Gastrointestinal Oncology and Endocrinology, University Medical Center Göttingen, Germany). These cells were derived from KPC mice^20^. KPC-Luc cells (KrasG12D; P53flox/flox; PDX-1-Cre; Luciferase positive) were provided by Dr. Cassian Yee and Prof. Craig Logsdon (M.D. Anderson Cancer Center, Houston, TX). These cells were derived from KPC mice (Kras^LSL-G12D^, Trp53^−/−^, and PDX-1-Cre) and transfected with enhanced firefly luciferase^21^. Both KPC and KPC-Luc cells were cultured in Dulbecco’s Modified Eagle Medium (DMEM; GIBCO) supplemented with 10% fetal bovine serum (FBS; GIBCO), 1% penicillin and streptomycin (pen/strep), and 1% L-glutamine. Cells were cultivated at 37 °C under a humidified atmosphere of 5% CO_2_.

### Cell proliferation assay

To measure cell proliferation in vitro, KPC and KPC-Luc cells were harvested and resuspended in complete DMEM. 1 × 10^5^ cells were plated in a T-25 flask. Cells were counted manually using a hemocytometer and the assay was repeated three times. To determine cell viability, 1 × 10^5^ KPC or KPC-Luc cells were plated in a final volume of 100 µl in a 96-well plate for 48–72 h. After this time, the MTS assay was performed: 20 µl per well of CellTiter 96^®^ AQueous One Solution Reagent (Promega) was added and the plate was incubated at 37 °C for 2 h. The amount of soluble formazan produced by cellular reduction of the tetrazolium compound [3-(4,5-dimethylthiazol-2-yl)-5-(3-carboxymethoxyphenyl)-2-(4-sulfophenyl)-2H-tetrazolium, inner salt; MTS] was measured by the absorbance at 490 nm using a plate reader (BioTek).

### Animals and orthotopic PDAC mouse model

All animal experimental procedures were performed in compliance with the European (2010/63/EU) and German regulations on Animal Welfare and were approved by the administration of Lower Saxony (LAVES; Nr. 33.19-42502-04–20/3527). All authors complied with the ARRIVE guidelines. Male C57BL/6 mice (12–15 weeks old) were kept under 12 h dark: light cycle with ad libitum access to food and water. For the orthotopic implantation, mice received 20–30 µl analgesia subcutaneously (s.c.) (Carprofen, Rycarfa 5 mg/kg; diluted 1:10 in 0.9% NaCl), and were anesthetized via inhalation of 2–3% isoflurane. Next, a small incision was made in the midline to access the pancreas, and 5 × 10^4^ KPC/Luc cells were injected into the head of the pancreas in 15 µl of Phosphate Buffered Saline (PBS) using an insulin syringe. Separate sets of sutures were used to close the peritoneum and skin (Ethicon, 4.0, 22 mm). Animals received analgesia for three days post-implantation and were monitored daily for weight loss and signs of distress following the surgery. Mice were sacrificed with an overdose of isoflurane, followed by cervical dislocation and the pancreatic tumors were excised and measured by a caliper. Blood was extracted by cardiac puncture. The spleen, peritoneal organs, lymph nodes, and lungs were excised and visually inspected for macroscopic metastases. The degree of metastatic spread was assessed by applying a metastasis score (Suppl. Table 1). For each organ, a number from 0 to 3 was given according to the number of metastases macroscopically present. The total metastases score was calculated by adding the individual organ scores. Each animal was scored individually.

### In vitro and in vivo bioluminescence imaging (BLI)

To confirm luciferase expression in the cells, either 1 × 10^5^ KPC or KPC-Luc cells were plated in a 96-well plate and incubated overnight. The next day, after washing the cells with PBS, d-luciferin solution was added to the wells (0.5–5 µM) and the bioluminescent signal was measured using the optical imaging scanner IVIS Spectrum (Perkin Elmer). For in vivo bioluminescence measurements, mice were injected with 150 mg/kg body weight (BW) of d-Luciferin (Promega) intraperitoneally (i.p.), immediately placed under isoflurane anesthesia (2–3%), and images of the abdomen acquired in an IVIS Spectrum. Tumor radiance was quantified in photons per second using the inbuilt Living Image software (Perkin Elmer). Gray-scale photographic images and bioluminescent color images were superimposed using the Living Image software overlay. To quantify emitted light, regions of interest were drawn over tumor regions, and total photon flux was determined.

### Preparation of blood and spleen samples

Blood was taken intracardially and directly placed in 1.5 ml tubes. 100 µl of blood was diluted 1:1 in PBS for flow cytometry analysis. The remaining blood was centrifuged at 1000*g* for 30 min for serum collection. The serum was kept at 20 °C until IgG measurement by ELISA. Spleens were mechanically dissociated into single cells and red blood cells (RBCs) were lysed by incubation in erythrocyte lysis buffer for 5 min at room temperature (RT). 10 ml of RPMI 1640 medium supplemented with 10 % FBS, 1 % pen/strep, 1 % l-glutamine (RPMI complete medium) was added to stop the lysis. Cells were then spun down at 300 g for 5 min, and resuspended in RPMI complete medium. To determine peptide-specific cytokine production, splenocytes were cultured in complete RPMI medium and stimulated with 20 µg/ml of a Luc peptide (LMYRFEEEL), which represents the immunodominant CD8 T cell epitope of Luc that is recognized by C57BL/6 mice^22^.

### Flow cytometry

Splenocytes and blood cells were stained with Zombie UV (Biolegend) as viability dye for 30 min, followed by staining in the presence of Fc block (CD16/32, Biolegend), and mouse monoclonal antibodies against CD45 (30-F11), CD3 (17A2), CD8a (53-6.7), CD4 (RM4-5), NK1.1 (PK136), CD19 (6D5), CD11b (M1/70), CD11c (N418), CD64 (X54-5/7.1), all from Biolegend. Antibodies were diluted 1:100 in FACS buffer (PBS + 2.5 % FBS) and cells were stained at RT in the dark for 20 min before acquisition using an LSRFortessa X-20 flow cytometer (BD Biosciences), and FACSDiva software (BD Biosciences). Data was analyzed using FlowJo software (version 10.8). For intracellular staining, following overnight stimulation with the Luc peptide and incubation in the presence of Brefeldin A (Biolegend) for the last 4 h, splenocytes were stained for Zombie UV, CD45, CD3, CD8a, CD4, as described above, then fixed for 20 min, permeabilized and stained for intracellular expression of interferon-γ (IFN-γ; XMG1.2, Biolegend) for 30 min in the dark on ice. Data acquisition was performed in the same way as for surface staining, as described above. Gating strategies are shown in Suppl. Figs. 1 and 2.

### Enzyme-linked immunosorbent assay (ELISA)

A standard sandwich ELISA was performed to determine the IFN-γ levels in the splenocyte culture supernatants (mouse Kit 88-7314-88, Invitrogen) and for the detection of IgG antibodies in the serum. Briefly, a 96-well ELISA plate was coated with the capture antibody (or Luc peptide; 10 µg/ml) in coating buffer and incubated at 4 °C overnight. Then the plate was washed three times with wash buffer (PBS—0.05 % Tween). After blocking for 1 h, the plate was washed and incubated with the samples and standards for 2 h at RT. The plate was then incubated with the biotinylated detection antibody for 1 h, followed by streptavidin-peroxidase incubation for 30 min, and finally, incubated with 3,3′,5,5′-Tetramethylbenzidine (TMB) substrate for approximately 10 min at RT. The reaction was stopped with 2 N H_2_SO_4_ and the absorbance was assessed with a plate reader (BioTek) at 450 nm.

### Immunofluorescence staining

Dissected tumors were fixed in 4% paraformaldehyde (PFA) in PBS overnight, then embedded in paraffin, and cut in tissue sections of 4 μm thickness using a microtome. Tumor sections were subjected to immunofluorescence staining for the detection of cytotoxic T cells and macrophages. For this purpose, slides were deparaffinized with xylol and rehydrated with increasing concentrations of EtOH, blocked with fish serum (37527, Invitrogen) for 20 min and incubated overnight with rabbit primary monoclonal antibody against CD8b (1:500 dilution, ab228965, Sigma) or polyclonal antibody against CD68 (1:500 dilution, ab125212, Sigma), followed by 1 h incubation with a goat anti-rabbit secondary antibody conjugated with Alexa Fluor 647 (1:200 dilution, a21244, Invitrogen), and counterstained with DAPI for 30 min for nuclei staining. Images were acquired with a Leica SP8 scanning confocal microscope using 20× magnification, 1024 × 1024 image resolution, 12-bit depth (approximately 6–12 images per tumor depending on the size). Images were analyzed by Fiji to quantify the number of positive cells. After automatic adjustment of the threshold, the background was eliminated by noise despeckling, the watershed mask was applied for cell segmentation, and finally, the number of cells was counted by the function “analyze particles”.

### Cytotoxicity assay

KPC and KPC-Luc cells were co-cultured with splenocytes from KPC-Luc tumor-bearing mice that developed for 13 days to measure the cytotoxicity of immune cells against the PDAC cells. For this purpose, cells were seeded (3 × 10^3^ cells/per well) on the day before co-culture. Splenocytes were cultured for 24 h, in the presence of Luc peptide (LMYRFEEEL, 5 µg/ml) and an adjuvant (R848, 2 µg/ml) or normal medium. After this period, 6 × 10^4^ splenocytes/per well were added to the KPC/KPC-Luc cells for 48 h. The confluence of the tumor cells was measured over time by a live-cell imaging system (Incucyte SX5, Sartorius). After 48 h of co-culture, a viability assay was performed according to the manufacturer’s protocol (CellTiter-Glo, Promega).

### Statistics

Statistical analysis was performed using GraphPad Prism software. All data are presented as mean ± SD. Unpaired Student’s t-test, or two-way ANOVA followed by Sidak’s was used for multiple comparisons. Differences between groups were considered significant at p < 0.05.

## Results

### Luc expression does not alter cell proliferation and viability in KPC cells

**Figure 1:**
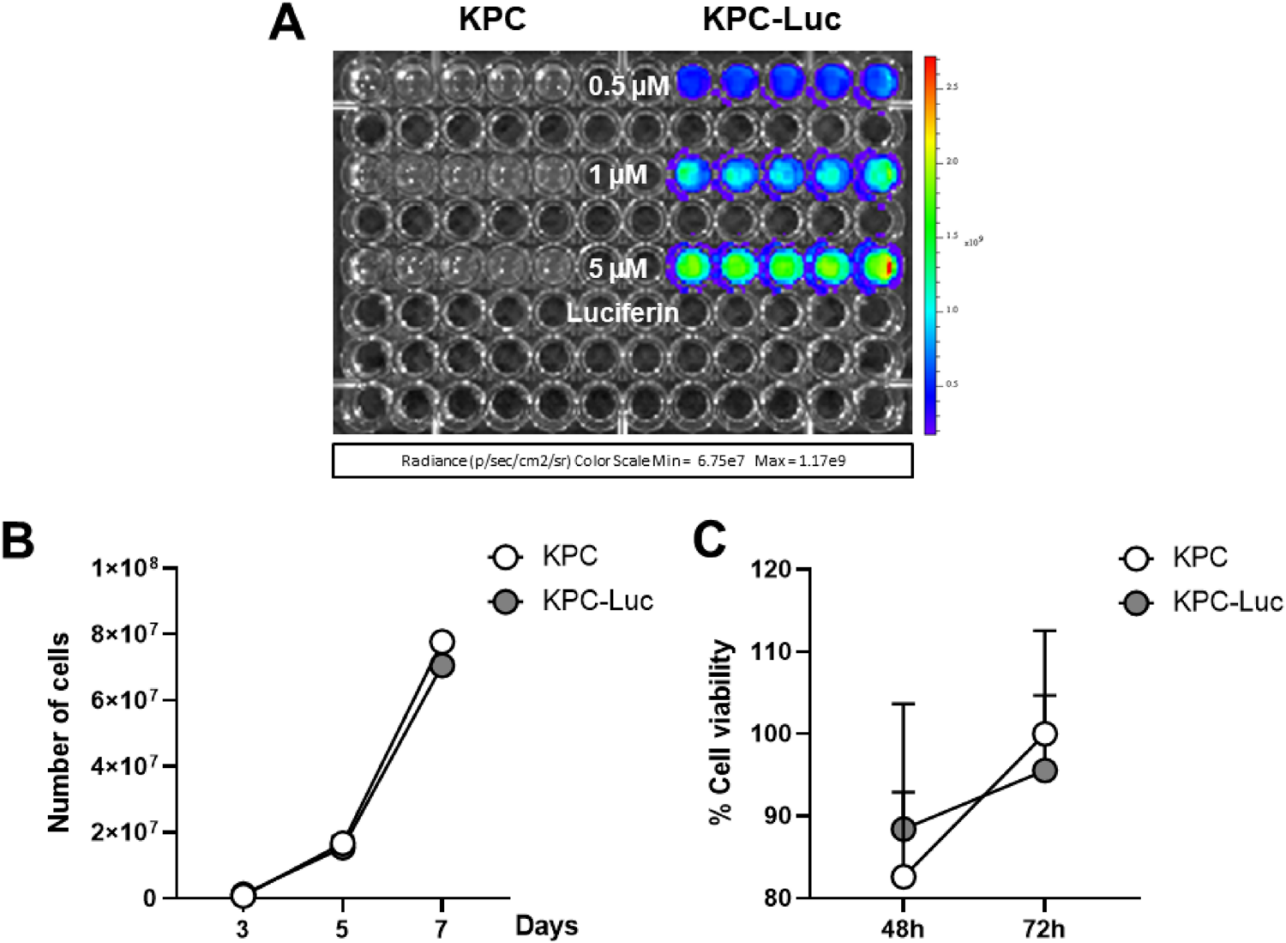
KPC-Luc cells show d-luciferin concentration-dependent bioluminescence and have similar proliferation rates and viability as KPC cells. (**A**) KPC-Luc cells show increasing dose-dependent bioluminescent signals after adding 0.5–5 μM of d-luciferin. (**B**) KPC and KPC-Luc cells display similar proliferation rates within 7 days of seeding of 1 × 10^5^ cells. (**C**) KPC and KPC-Luc cells reveal similar viability 48 and 72 h after seeding 1 × 10^5^ cells, assessed by MTS assay. Four to five replicates per group were used. Data is presented as mean ± SD.

Luc expression in the murine KPC-Luc tumor cells was assessed by adding increasing concentrations of d-luciferin to the wells. After 5 min, KPC-Luc cells showed intensification of the bioluminescence signals in a dose-dependent way (Fig. 1A). KPC control cells, that do not express the Luc gene, were used as a negative control and did not show any signs of bioluminescence in response to d-luciferin in vitro.

To investigate whether the expression of Luc affects the proliferation kinetics of KPC cells, we assessed and compared the growth rates of KPC and KPC-Luc cells in vitro. Both cell lines were seeded at the same density (1 × 10^5^ cells) in T-25 flasks and the number of cells was manually counted after 3, 5, and 7 days. Our data show that KPC and KPC-Luc cells have comparable proliferation rates in vitro (Fig. 1B). Using the MTS viability assay, we demonstrated that both cell lines had similar percentages of viability at both 48 and 72 h after seeding in a 96-well plate (Fig. 1C).

### Luc expression inhibits tumor development by KPC cells in vivo

**Figure 2:**
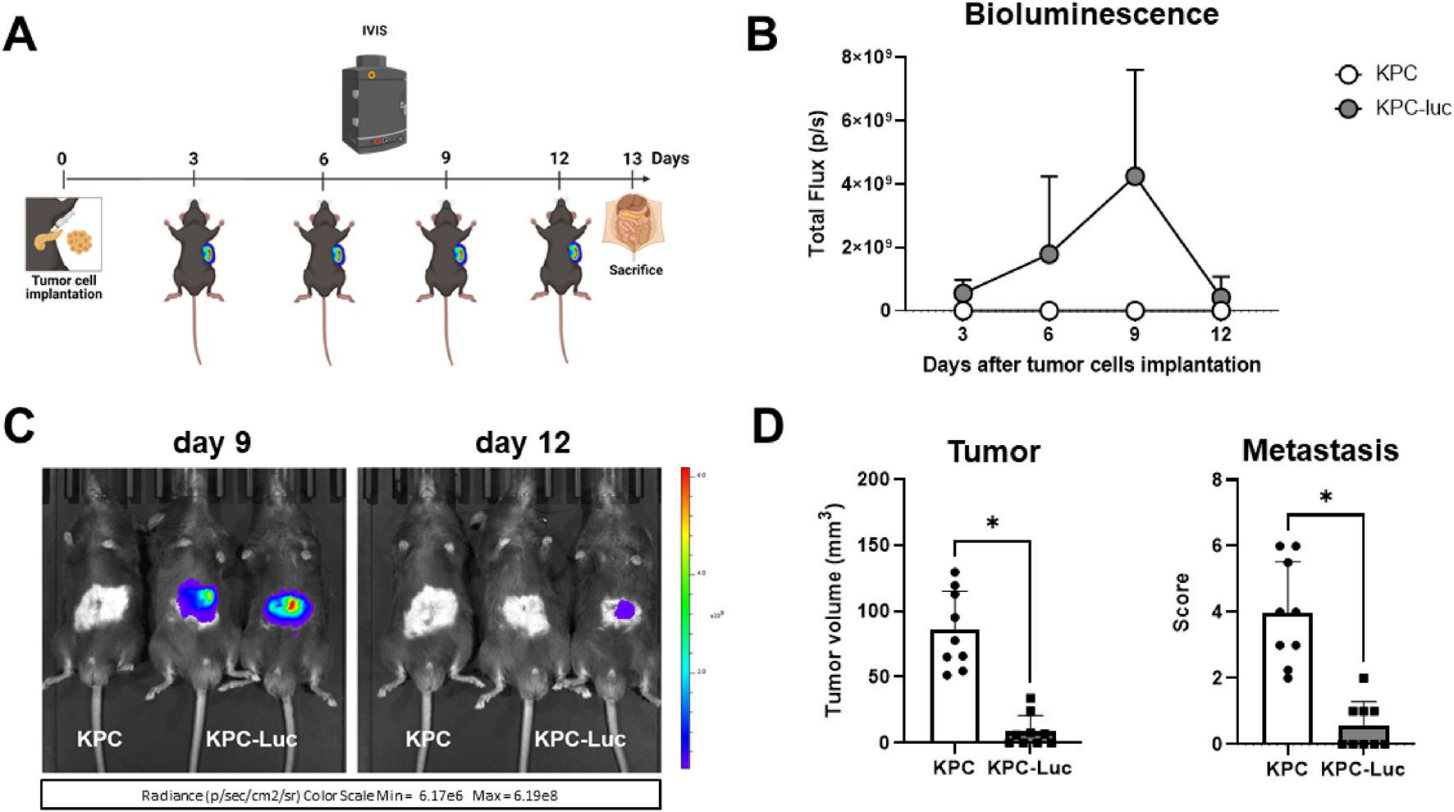
KPC-Luc tumors regress 13 days after orthotopic cell implantation in mice. (**A**) Scheme of the in vivo workflow. After injection of 5 × 10^4^ cells tumor cells in the head of the pancreas, tumor growth was monitored by bioluminescence imaging using an IVIS Spectrum every third day. Mice were sacrificed on day 13 post-cell implantation. (**B**) In KPC-Luc tumors the bioluminescent signal increased progressively until day 9 post-implantation, then it drastically dropped on day 12. Bioluminescence intensity is presented as photons/second. (**C**) Representative images of KPC non-bioluminescent and KPC-Luc bioluminescent tumors on day 9 and 12 after tumor cell implantation are shown, demonstrating a decrease in the bioluminescence signal of KPC-Luc tumors by day 12. (**D**) KPC-Luc tumor mice presented significantly smaller primary tumors at day 13, and resulted in a lower mean score for metastasis, compared to KPC tumor mice. Data is presented as mean ± SD. Unpaired t-test *p < 0.0001; n = 9.

The impact of Luc expression on KPC tumorigenicity and tumor growth was evaluated in vivo. For this, either 5 × 10^4^ KPC or KPC-Luc cells were injected into the head of the pancreas of C57BL/6 male mice and BLI was performed every third day after surgery to monitor tumor growth (Fig. 2A). All mice that were implanted orthotopically with KPC-Luc cells showed bioluminescent signals from the third day post-surgery onwards, which increased until day 9. On day 12 post-cell implantation, the BLI signals drastically dropped (Fig. 2B,C). KPC tumor-bearing mice injected with d-luciferin did not show any bioluminescence, as expected (Fig. 2C). All animals were sacrificed on day 13 after cell implantation and tumors were extracted. We determined that KPC-Luc tumors were significantly smaller (8.73 ± 12.2 mm^3^) than KPC tumors (85.8 ± 29.4 mm^3^; Fig. 2D). In contrast to KPC-Luc implanted mice, we observed a higher number of metastasis in KPC-tumor-bearing mice, at sites of adjacent organs such as liver and kidneys, as well as at the mesentery. The final metastasis score of KPC-Luc tumor-bearing mice was significantly lower (0.55 ± 0.7) than the one assessed in KPC tumor mice (3.97 ± 1.5; Fig. 2D).

### Mice with KPC-Luc tumors display altered immunophenotype and their splenocytes show an increased immune reaction against Luc peptide in vitro

**Figure 3:**
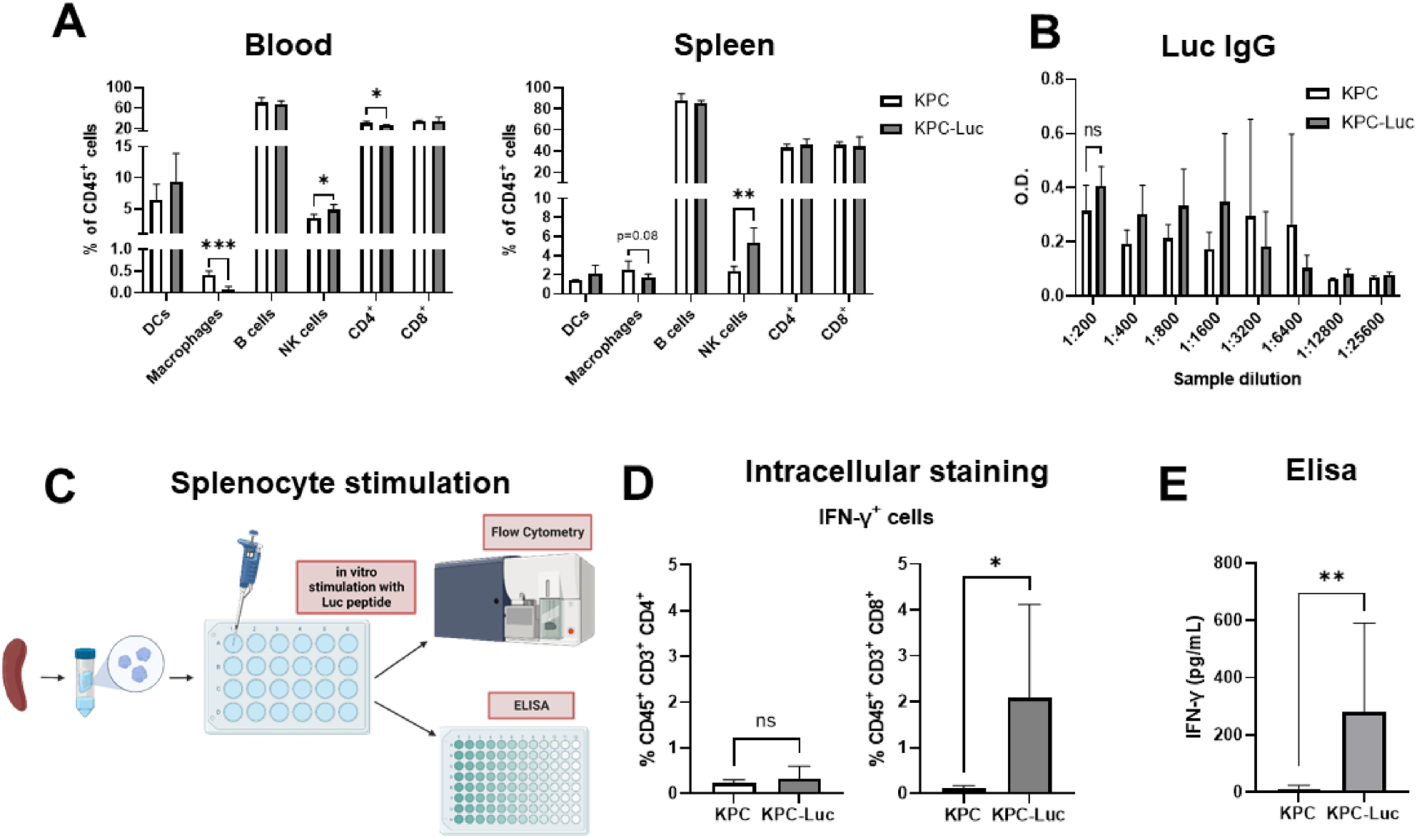
KPC-Luc tumor-bearing mice show an anti-tumor immune profile 13 days after tumor implantation. (**A**) Spleen and blood samples were analyzed by flow cytometry for the presence of dendritic cells (DCs), macrophages, B cells, natural killer (NK) cells, CD4^+^ and CD8^+^ T cells; n = 5. (**B**) IgG levels in serum were similar between KPC and KPC-Luc tumor mice, measured by ELISA. (**C**) Splenocytes were isolated from the spleens of KPC and KPC-Luc tumor-bearing mice and further stimulated with 20 µg/ml Luc peptide (LMYRFEEEL). Levels of IFN-γ were analyzed by flow cytometry and ELISA. In vitro, stimulation of the splenocytes led to (**D**) an increased number of CD8^+^ IFN-γ^+^ cells from KPC-Luc tumor-bearing mice, compared to KPC mice, evaluated by intracellular staining, and (E) increased levels of IFN-γ in the supernatant of splenocytes from mice that developed KPC-Luc tumors, measured by ELISA; n = 9; Data is presented as mean ± SD. Unpaired t-test between the groups. *p < 0.05, **p < 0.01, ***p < 0.001. ns: not significant.

Because KPC-Luc cells only had small tumors at dissection, it was not possible to analyze the immune cells within the tumor microenvironment by flow cytometry. Due to the very limited amount of tumor tissue, we performed immune profiling in the peripheral blood and the spleen of tumor-bearing mice. At 13 days of tumor growth, we determined a decrease in the number of macrophages (CD45^+^ CD11b^+^ CD64^+^ cells), and CD4^+^ T cells (CD45^+^ CD3^+^ cells) in the blood of KPC-Luc tumor-bearing mice, compared to KPC tumor mice (Fig. 3A). In addition, in the blood and the spleen of KPC-Luc mice the number of natural killer (NK) cells (CD45^+^ CD3^−^ NK 1.1^+^ cells) increased, in comparison to KPC tumor mice (Fig. 3A).

Humoral response against Luc was evaluated by determining the levels of IgG antibodies in the serum of tumor-bearing mice. Our data showed no differences in the quantity of IgG antibodies measured by ELISA between KPC and KPC-Luc tumor mice (Fig. 3B), indicating that B cell response did not contribute to the immunogenicity against Luc in the tumors.

We hypothesized that the inhibition in tumor development and metastasis demonstrated by KPC-Luc cells might be due to an immune response against luciferase. To confirm this, splenocytes from KPC and KPC-Luc tumor mice were isolated and re-stimulated in vitro with a peptide that represents the immunodominant CD8 T cell epitope of Luc that is recognized by C57BL/6 mice^22^ (Fig. 3C). Using intracellular staining, we observed that the number of activated CD8^+^ T cells (CD45^+^, CD3^+^cells) that express IFN-γ was higher in splenocytes from KPC-Luc tumor mice than in the KPC tumor group. No differences were found in the number of CD4^+^ IFN-γ^+^ cells (CD45^+^, CD3^+^) between the groups (Fig. 3D). Furthermore, Luc-specific IFN-γ response was significantly higher in the KPC-Luc tumor mice, than in the KPC tumor group, as measured in the supernatant of splenocytes by ELISA (Fig. 3E).

### KPC and KPC-Luc cells develop tumors of similar sizes at an early stage of tumor progression

**Figure 4:**
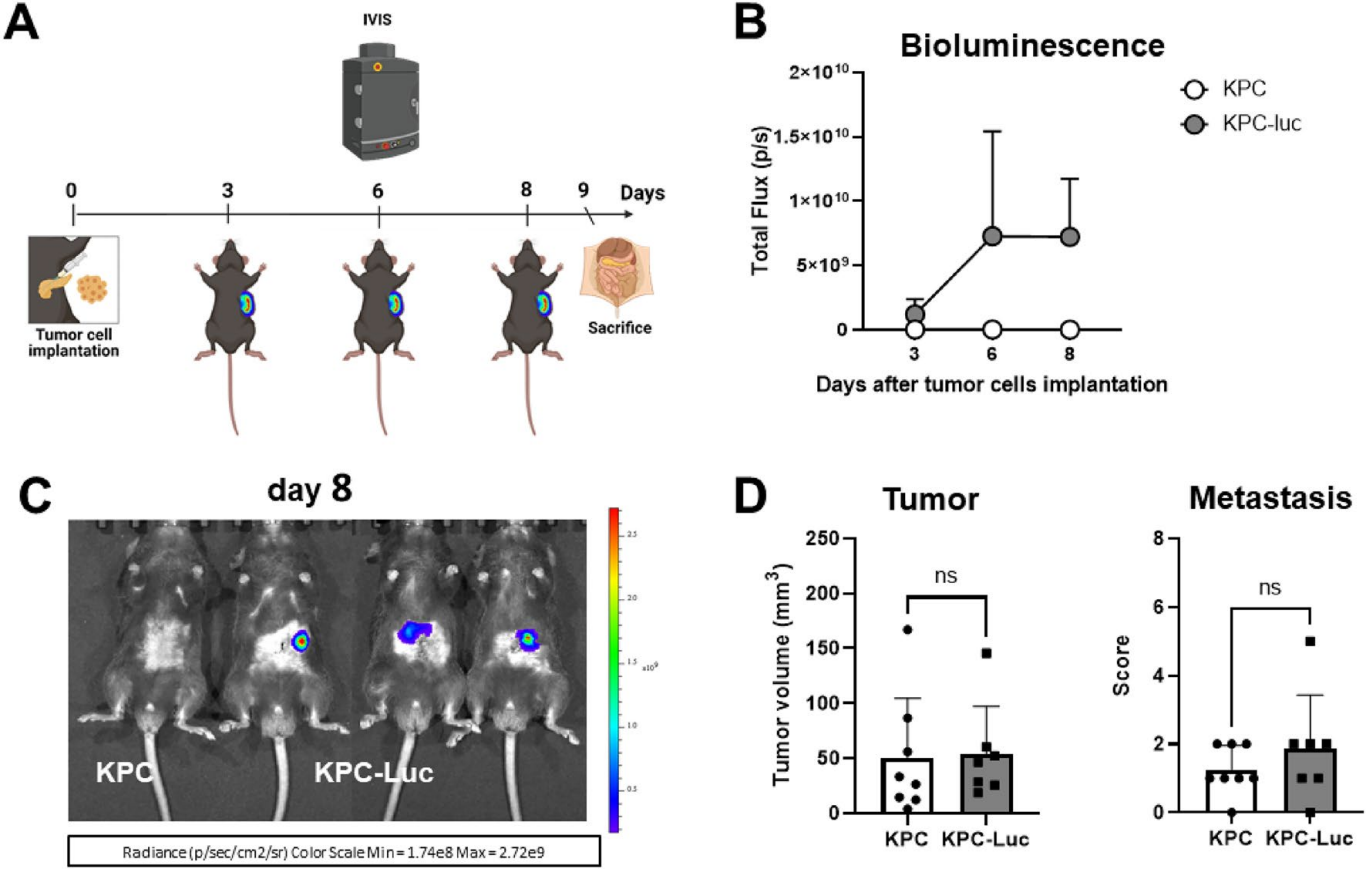
KPC and KPC-Luc cells develop tumors with comparable volumes in mice 9 days after cell implantation. (**A**) After the injection of 5 × 10^4^ tumor cells in the head of the pancreas, tumor growth was monitored by BLI imaging using the IVIS Spectrum every third day. Mice were sacrificed on day 9 post-tumor implantation. (**B**) In KPC-Luc tumors, the bioluminescent signal was present until day 8, without any significant decrease. (**C**) Representative images illustrating bioluminescent signals over the tumor areas on day 8 after cell implantation. (**D**) Sizes and metastatic scores of KPC and KPC-Luc tumors assessed 9 days after orthotopic implantation. Note the similar tumor volumes and comparable metastatic sores at this time point. Data is presented as mean ± SD. Unpaired t-test; ns: not significant; n = 9.

We demonstrated that by day 12 after cell-implantation, bioluminescence intensity decreased, and on day 13 KPC-Luc tumors were much smaller or even absent when compared to KPC tumors. Furthermore, 13 days following cell implantation, mice displayed an immune response against Luc. We hypothesized that at an earlier time point, when the bioluminescent signal was stable, KPC-Luc tumors were still developing before an anti-Luc immune response occurred. To examine this hypothesis, we induced KPC and KPC-luc tumors as before but sacrificed the mice earlier at day 9 after cell implantation (Fig. 4A). As expected, KPC-Luc injected animals showed bioluminescence from day 3 post-surgery onwards (Fig. 4B). By the 8^th^ day the signal was still measurable in all animals (Fig. 4C). Interestingly, at day 9 KPC and KPC-Luc implanted mice had developed tumors of a similar size, displayed comparable metastatic spread in the liver and showed similar scores for metastasis (Fig. 4D). These data confirmed that at early time points after implantation, KPC-Luc cells develop solid tumors in the pancreas, but later, due to a spontaneous immune reaction, tumors start to regress, along with the number of metastasis, as we demonstrated for day 13 post surgery.

### Mice with KPC-Luc tumors display altered immunophenotype and no immune reaction against Luc peptide in vitro at an early stage of tumor progression

**Figure 5:**
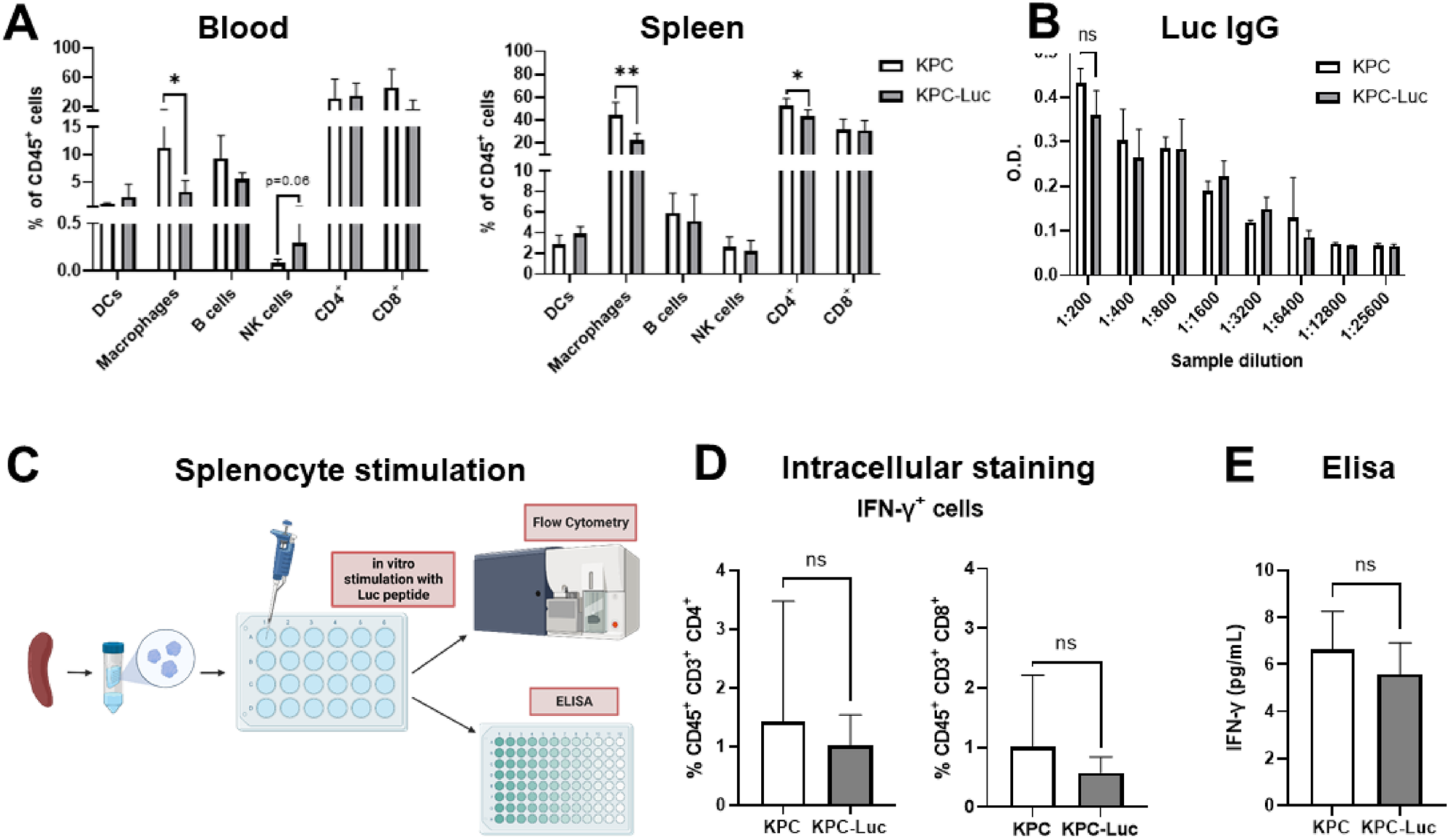
KPC-Luc tumor-bearing mice have a higher number of macrophages and T helper cells but do not show an immune response against Luc peptide 9 days after tumor implantation. (**A**) Spleen and blood samples of tumor-bearing mice were analyzed by flow cytometry for dendritic cells (DCs), macrophages, B cells, NK cells, CD4^+^, and CD8^+^ T cells; n = 4–6. (**B**) Serum IgG levels were similar between KPC and KPC-Luc tumor mice, measured by ELISA. (**C**) Splenocytes were isolated from the spleens of KPC and KPC-Luc tumor-bearing mice. The cells were stimulated with 20 µg/ml of Luc peptide (LMYRFEEEL) in vitro and levels of IFN-γ were analyzed by flow cytometry and ELISA. In vitro stimulation of the splenocytes did not show any differences in (**D**) the number of IFN-γ^+^ splenocytes evaluated by intracellular staining, or in (**E**) the levels of IFN-γ in the splenocyte supernatant measured by ELISA; n = 4–8; Data is presented as mean ± SD. Unpaired t-test; *p < 0.05, **p < 0.01; ns: not significant.

To compare the immune profile of KPC and KPC-Luc mice 9 days after cancer cell implantation, blood and spleen of tumor-bearing mice were collected for flow cytometry analysis. As previously observed in the group that was sacrificed 13 days after surgery, we found a decrease in the number of macrophages (CD45^+^ CD11b^+^ CD64^+^ cells) in the blood and spleen of KPC-Luc tumor mice, compared to KPC tumors as determined. Moreover, we observed a decreased number of CD4^+^ T cells (CD45^+^, CD3^+^cells) in the spleen of KPC-Luc mice, compared to the KPC group. In the blood of KPC-Luc tumor-bearing mice, there was a tendency to a higher number of NK cells, but without reaching significance (Fig. 5A). There were no differences in IgG antibody levels in the serum of KPC and KPC-Luc cells implanted groups (Fig. 5B).

To confirm the involvement of CD8^+^ T cells in tumor regression, we analyzed the activation response of T cells to Luc peptide, using splenocytes from tumor-bearing mice (Fig. 5C). 9 days post-cell implantation, the number of CD8^+^ IFN-γ^+^ cells, or the levels of IFN-γ in the supernatants did not differ in the two groups (Fig. 5D,E), which corresponds to the appearance of similar tumor sizes in the KPC and KPC-Luc cell implanted mice. These data indicate that at this time point of tumor development (9 days) the immune reaction has not yet impacted the growth rate of tumors, due to low cytotoxic T cell activation. The much stronger immune response, that was observed at a later stage of tumor development, suggests a direct impact on the partial or complete tumor regression. In addition, the cytotoxicity of immune cells from KPC-Luc tumor-bearing mice against KPC and KPC-Luc cells was analyzed. In comparison to KPC, KPC-Luc cells showed significantly reduced confluence and viability when co-cultured with splenocytes from KPC-Luc tumor-bearing mice for 48 h (Suppl. Fig. 3).

### KPC-Luc tumors show higher infiltration of T cells than KPC tumors

**Figure 6:**
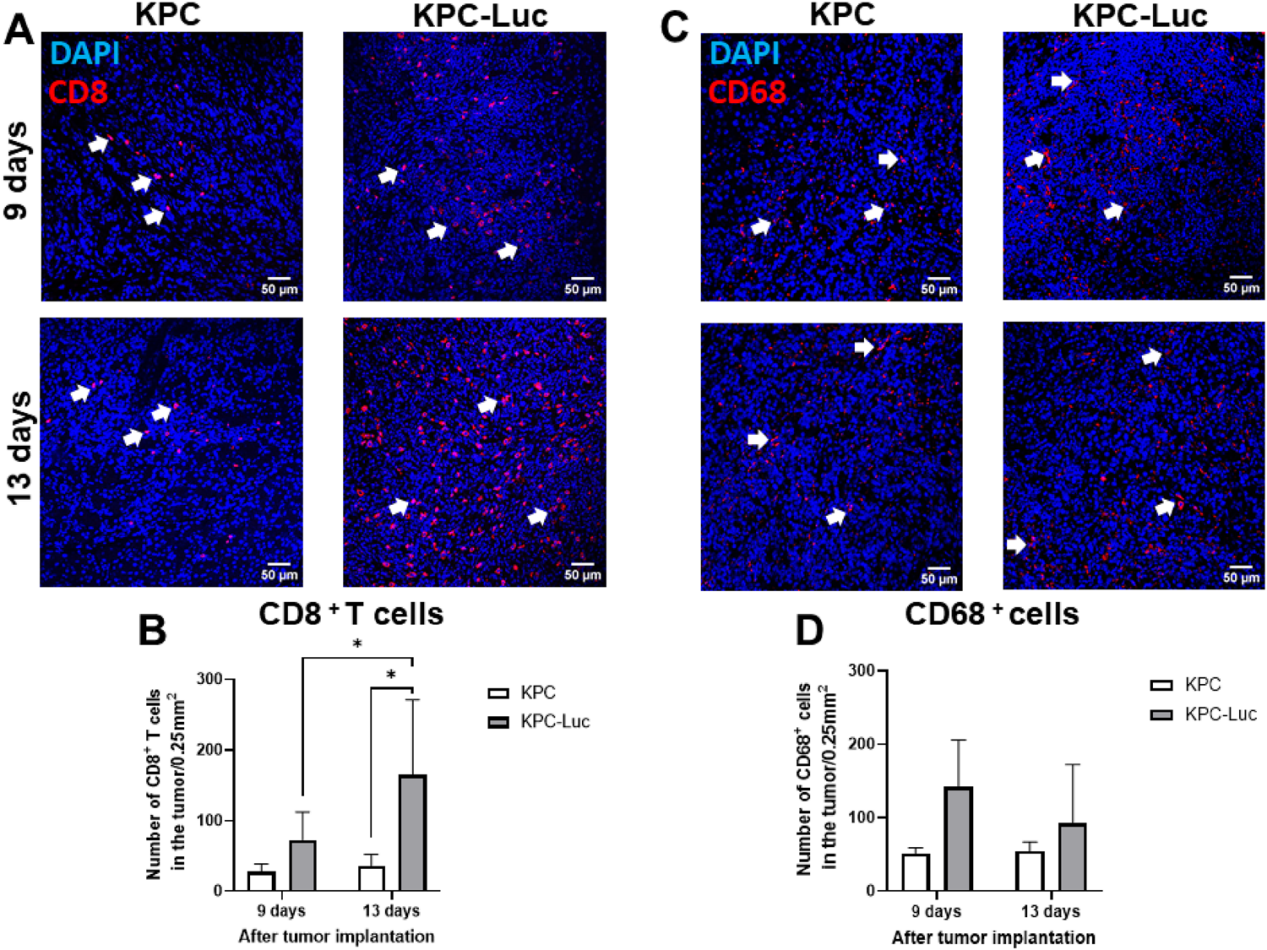
KPC-Luc tumors have increased immune cell infiltration when compared to KPC tumors. Immunofluorescence staining of immune cell infiltration in the tumor (red). Nuclei are stained by DAPI (blue). White arrows point to positive stainings. (**A**) Representative images of immunofluorescence staining of KPC and KPC-Luc tumors 9 and 13 days after cell implantation, showing infiltration of CD8^+^ T cells. (**B**) Quantitative analysis depicted a significantly increased number of CD8^+^ T cells infiltrated in the tumors of KPC-Luc cells implanted mice, when compared to KPC cells implanted mice, at 13 days of tumor growth, but not at 9 days. Moreover, in the KPC-Luc group, tumor-infiltrated CD8^+^ T cells increased between days 9 and 13 after cell implantation; n = 3. (**C**) Representative images of immunofluorescence staining of KPC and KPC-Luc tumors 9 and 13 days after cell implantation, showing infiltration of CD68^+^ cells (macrophages). (**D**) Quantitative analysis showed no significant differences in the number of CD68^+^ cells infiltrated in the tumor of KPC and KPC-Luc implanted mice at 9 or 13 days of tumor growth; n = 3; Data is presented as mean ± SD. Two-way ANOVA followed by Sidak’s multiple comparisons; *p < 0.01. Scale bars in (**A**) and (**C**) represent 50 µm.

Our next step was to evaluate how Luc expression in cancer cells can influence immune cell infiltration in pancreatic tumors. For this purpose, we analyzed the presence of cytotoxic T cells (CD8^+^) and macrophages (CD68^+^) in primary tumor samples by immunofluorescence. We observed a significant increase in the number of CD8^+^ T cells in the KPC-Luc tumors grown over 13 days, compared to KPC tumors, but not in tumors from mice sacrificed at 9 days. Moreover, immunofluorescence staining showed that in the KPC-Luc group, T cell infiltration was higher at 13 days of tumor development compared to day 9 tumors, indicating increasing immunogenicity in the tumors over time (Fig. 6A and B). This increase in T cell infiltration over time was not observed in the KPC tumor sections (Fig. 6A). On the other hand, the number of CD68^+^ macrophages in the tumor was not significantly altered in any group at any time point analyzed (Fig. 6C and D), despite showing a decrease in blood and spleen samples. These data confirm that an anti-Luc response was mediated mainly by CD8^+^ T cells.

### KPC-Luc tumors regress and do not regrow after 70 days

**Figure 7:**
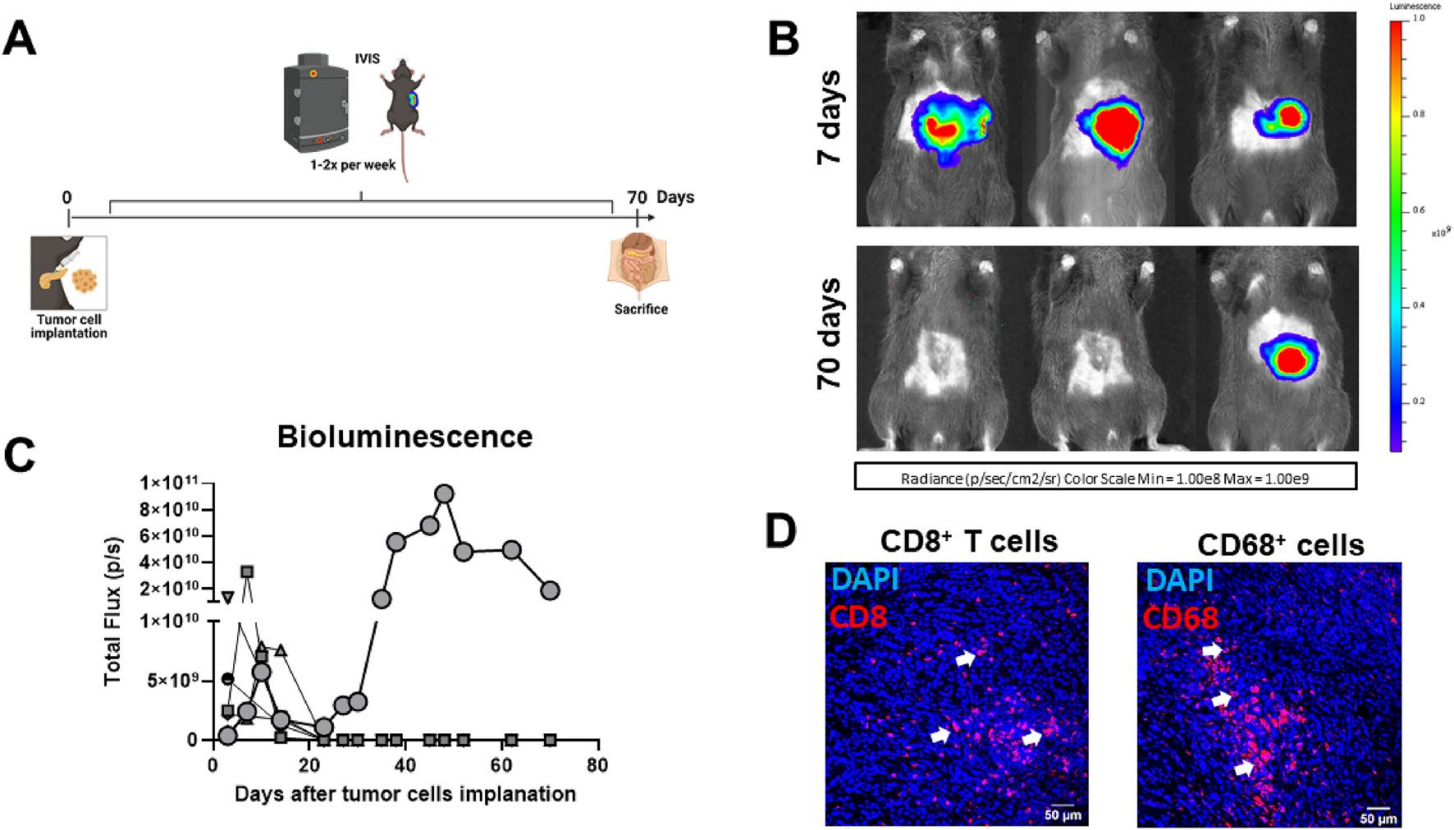
After regression, KPC-Luc tumors do not regrow. (**A**) Mice were injected orthotopically with 5 × 10^4^ KPC-Luc cells and monitored for tumor growth once or twice per week by IVIS Spectrum. 70 days after cell implantation, mice were sacrificed and analyzed for the presence of tumors. (**B**) Representative images of bioluminescent tumors are shown 7 and 70 days after tumor cell implantation. The majority of mice lost the bioluminescence signal by day 70. (**C**) Graph showing bioluminescence signals of 5 individual mice over time. The signal was present until the second-week post-implantation before it decreased. In four out of five mice, no bioluminescence signal could be detected from day 3 to 70 after cell implantation. Only one mouse had a constant increase of signal from 20 days after tumor cell injection that persisted until the day of sacrifice. (**D**) Immunofluorescence staining showed infiltration of CD8^+^ T cells and CD68^+^ cells in a representative section of a tumor obtained 70 days after KPC-Luc implantation. White arrows point to positive staining of the immune cells. Scale bars in (**D**) represent 50 µm.

Next, we investigated if KPC-Luc tumors continue to regress over time or regrow in due course. For this purpose we injected 5 × 10^4^ KPC-Luc cells in the head of the pancreas of five C57BL/6 mice and monitored tumor growth by BLI once or twice per week for a period of 70 days (Fig. 7A). All five examined mice showed a high intensity of bioluminescence over the tumor area by the first week and, as expected, the signal dropped over the second week. Following this decrease of bioluminescence in all mice, only one animal showed tumor regrowth by BLI at 20 days after the surgery. On day 70, the bioluminescence signal was still present in this mouse (Fig. 7B,C), which developed a rather large primary KPC-Luc tumor of 892.2 mm^3^. Immunofluorescence staining of this pancreatic tumor (Fig. 7D) revealed a substantial infiltration of CD8^+^ T cells (average of 204.1 ± 90 positive cells per 0.25 mm^2^) and CD68^+^ cells (317.5 ± 169.1 per 0.25 mm^2^). Moreover, in the four mice without bioluminescence signals no tumor was found in the pancreas and no visible metastasis were present upon autopsy.

## Discussion

Imageable reporters, such as Luc have been widely applied in in vivo cancer studies and are essential for accurately tracking cancer development and progression. These models can demonstrate in real-time the antitumor and antimetastatic response of novel therapeutic agents against malignancies by BLI over time and are thus valuable tools in preclinical research^13,23,24^. In this study, we demonstrate for the first time in PDAC that the expression of luciferase in murine KPC-Luc cells, a widely used reporter for PDAC growth in mouse models, induces a potent immune response between day 9 and 13 after orthotopic tumor cell implantation in immunocompetent mice, that results in permanent tumor regression up to 70 days.

Although in vitro proliferation of KPC and KPC-Luc cells were comparable, we show that 13 days after cell implantation KPC-Luc tumors present significantly smaller volumes than KPC tumors and fewer metastases in the liver. The effect of luciferase expression in tumor cells on cancer development has been controversial, as in most studies Luc did not impact tumor development. Likewise, KPC-luc cells were used previously for tumor implantation in the investigation of immunotherapy for PDAC^21,25,26^. In contrast to our results, these studies reported an increased tumor volume by BLI from 7 to 28 days in untreated female C57BL/6 mice or male albino C57BL/6 mice following injection of 2 × 10^5^–1 × 10^6^ KPC-Luc cells in the tail of the pancreas. We hypothesize that the influence of luciferase expression in inducing an immune reaction and inhibiting tumor progression might be partially model-dependent. In our PDAC mouse model, the orthotopic implantation was performed in the head of the pancreas and in male mice, whereas the other studies used different genders, different pancreas locations and/or higher amounts of cells for implantation. Considering this, it is possible that our contrasting results could be due to differences in these methodological approaches, highlighting the importance of selecting an appropriate tumor mouse model for the assessment of tumor progression by BLI imaging. Moreover, in line with our observations, some studies using cancer cells of different tumor entities reported that Luc expression altered tumor progression in vivo. For example, mice with bioluminescent GL261 glioma tumors showed longer survival in comparison to mice bearing their non-bioluminescent control tumors^27^. Lewis lung carcinoma (LL/2) cells transduced with dTomato and luciferase, decreased tumorigenicity, compared to non-transduced cells^18^. Furthermore, in our study, the mouse that had a KPC-Luc tumor for 70 days did not show any signs of distress at this period, whereas in studies using orthotopic KPC cell implantation, mice do not commonly survive for more than 30 days^21,28^, which indicates that KPC-Luc tumors are not as aggressive as KPC tumors, as mice present prolonged survival when implanted with bioluminescent cells.

Our study provides evidence of the role of CD8^+^ T cells in exerting an anti-tumor response against luciferase that culminated in tumor regression. Firstly, we showed that Luc expression in KPC-Luc cells induces a potent immune response, indicated by a higher number of infiltrated cytotoxic T cells in the tumors, and NK cells in the blood and spleen of KPC-Luc tumor-bearing mice in comparison to KPC tumor-bearing mice. Such an immunophenotype of KPC-Luc tumor samples corresponds to an anti-tumoral profile of the immune cells observed by others^29,30^. Since KPC tumors are poorly immunogenic, and are known for evading immunosurveillance^31^, the immune responses we found in KPC-Luc-bearing mice between day 9 and 13 are surprising and most likely explain the inhibition of tumor growth we observed on day 13 after cell implantation. It is well-known that cytotoxic CD8^+^ T cells are the most powerful effectors in the anticancer immune response^32^. Along with NK cells, they promote tumor regression via the release of the cytolytic content of their granules such as perforin, granzymes, and the cytokine IFN-γ^33^. Our data correlates with similar observations in other tumor mouse models, such as for glioblastoma, where the injection of CT2A-Luc cells in the brain drastically increased the number of T cells locally, compared with wild-type controls^34^, and murine lung carcinoma, as seen by increased tumor-infiltrating lymphocytes (TILs) and decreased tumor-induced myeloid-derived suppressor cells (MDSCs) in Luc-expressing tumors, in comparison to non-Luc tumors^18^.

Secondly, we observed an increase in specific CD8^+^ T cell response against Luc peptide in splenocytes derived from KPC-Luc tumors on day 13 after induction, when compared to splenocytes from KPC tumors. A similar response was reported by Limberis et al.^22^, or in mice bearing 4T1Luc-expressing tumors where there was a higher IFN-γ response to the dominant cytotoxic T lymphocyte (CTL) epitope of Luc, compared to mice bearing non-Luc tumors^16^. Likewise, mice submitted to pcDNA3.1‑Fluc vaccination and later implantation of Luc-expressing CT26/Luc cells showed higher levels of IFN-γ in draining lymphoid cells and its secreted supernatant^19^, suggesting a specific response to the FLuc protein. In addition, we found higher cytotoxicity of splenocytes from KPC-Luc tumor-bearing mice against KPC-Luc cells, in comparison to KPC, suggesting a memory response by the immune cells. The expression of foreign proteins, such as luciferase in cancer cells could act as a foreign antigen that is targeted by the immune system, thus altering the adaptive immune response. Consequently, the use of such imaging techniques might interfere with the results from immunotherapeutic studies, since the immunogenic anti-tumor responses may be augmented.

Interestingly, regression of KPC-Luc tumors starts 9 days after orthotopic cell injection, as KPC and KPC-Luc implanted mice still displayed similar tumor volumes at this time point. Coincidentally, no specific T cell activation was observed after ex-vivo stimulation of splenocytes from both KPC and KPC-Luc tumor groups with Luc peptide. Similar observations were reported in a breast cancer model, where only a weak Luc-specific production of IFN-γ was induced 9 days after tumor cell implantation in BALB/c mice, but which increased to significant levels by day 23^16^. Our data suggest that at an early stage of tumor development, and prior to immune system activation, KPC-Luc cells can develop solid tumors in the pancreas. However, a later response against the presence of a foreign antigen in the tumor cells, in our case luciferase, led to permanent tumor regression, as seen in the mice sacrificed 70 days after cell implantation, that did not present tumors anymore. Another study reported a comparable effect of green fluorescent protein (GFP) in 4T1 breast cancer cells, where 11 days after inoculation in BALB/c mice, tumor rejection was observed followed by a robust GFP-specific CD8 + T cell response^35^. The complication with xenobiotic proteins can be reversed by genetically engineered mice tolerant to reporters, where normal tumor development is observed^17,36^, offering an alternative for the preclinical evaluation of traceable tumors.

In the blood and the spleen of KPC-Luc tumor-bearing mice, we observed a reduction in CD4^+^ T cells and macrophages, which could be a possible mechanism in the tumor regression effect mediated by Luc. An anti-tumor reaction can be limited by the accumulation of CD4^+^ regulatory T cells (Tregs), which have been shown to suppress effector T cell response^37,38^. However, in our study, we did not use specific markers for the evaluation of Tregs (CD25 and FoxP3), and therefore we cannot confirm that it was this subset of CD4^+^ T cells that was decreased in the samples of KPC-Luc tumor mice. Similarly, macrophages are abundant in the tumor microenvironment, playing a pro-tumoral role, by preventing tumor cell attack by cytotoxic T cells and NK cells and enhancing tumor cell invasion^39^. A decrease in immunosuppressive cells allows higher infiltration of cytotoxic T cells in the tumor, thus leading to cancer cell death^32,40^. Other studies reported reduced Tregs and myeloid precursor cell populations with tumor-promoting capacity in luciferase-expressing tumor mice^18,27^.

Overall, our study shows for the first time that Luc expression by KPC cells induces anti-tumor immunity, leading to tumor regression in the second week post-cell implantation. Therefore, data from studies using Luc as an imaging reporter in tumor cells to monitor tumor growth must be carefully interpreted. Researchers must consider these results when choosing a suitable in vivo mouse pancreatic cancer model in combination with BLI imaging, especially for the investigation of immunotherapy efficacy against PDAC.

### Supplementary Information

Supplementary Information.

## Data Availability

The data presented in this study are available on request from the corresponding author.
