# Supplementary material for: KPC-luciferase-expressing cells elicit an anti-tumor immune response in a mouse model of pancreatic cancer
Source: Sci Rep. 2024 Jun 13;14:13602. doi: 10.1038/s41598-024-64053-0 (PMC11169258; doi:10.1038/s41598-024-64053-0)
Supplement: Supplementary file 1 — Supplementary Information. [file 41598_2024_64053_MOESM1_ESM.docx]

**Supplementary information**

**KPC-luciferase-expressing cells elicit an anti-tumor immune response in a mouse model of pancreatic cancer**

Daniele Pereira Ferrari^1^, Fernanda Ramos-Gomes^1^, Frauke Alves^1,2,3^; M. Andrea Markus^1,*^

^1^ Translational Molecular Imaging, Max-Planck-Institute for Multidisciplinary Sciences, Hermann Rein ‑Straße 3, 37075 Göttingen, Germany

^2^ Institute of Diagnostic and Interventional Radiology, University Medical Center Göttingen, Robert-Koch-Str. 40, 37075 Göttingen, Germany

3 Department of Haematology and Medical Oncology, University Medical Center Göttingen, Robert-Koch-Str. 40, 37075 Göttingen, Germany

*corresponding author: markus@mpinat.mpg.de

**Suppl. Table 1. Metastases scoring.** Score sheet for the assessment of metastases appearance in different organs.


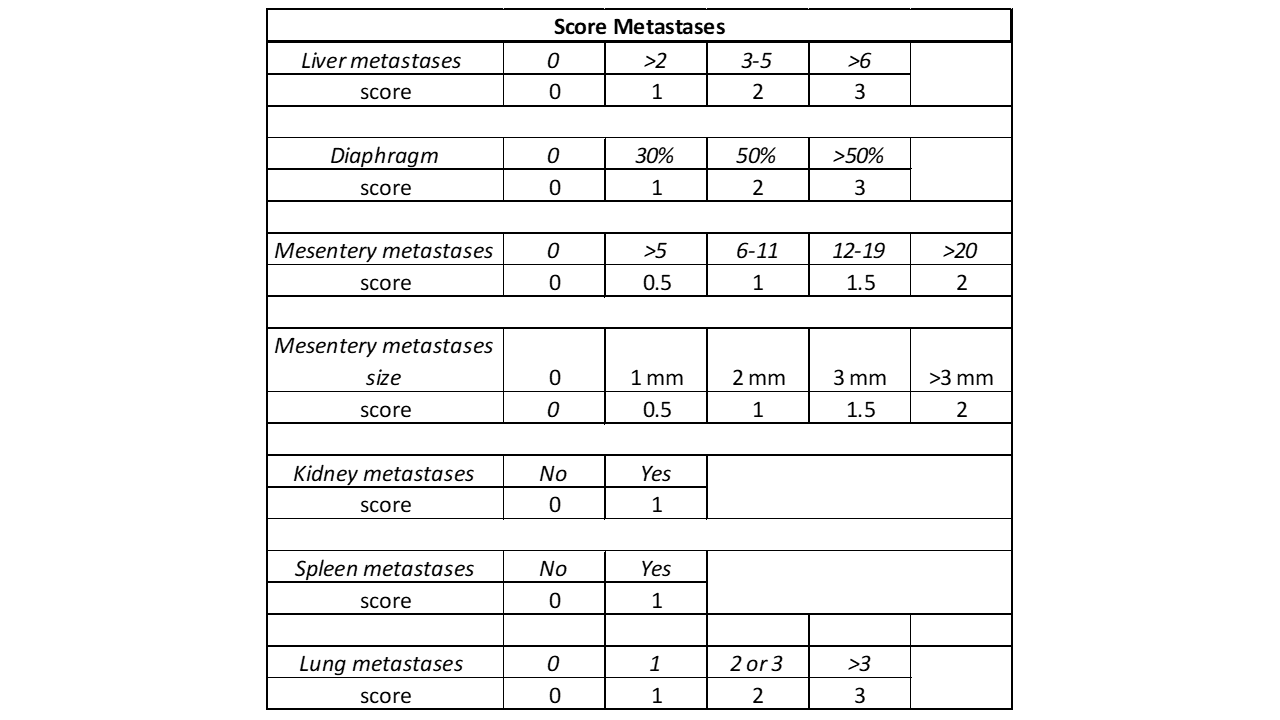


**
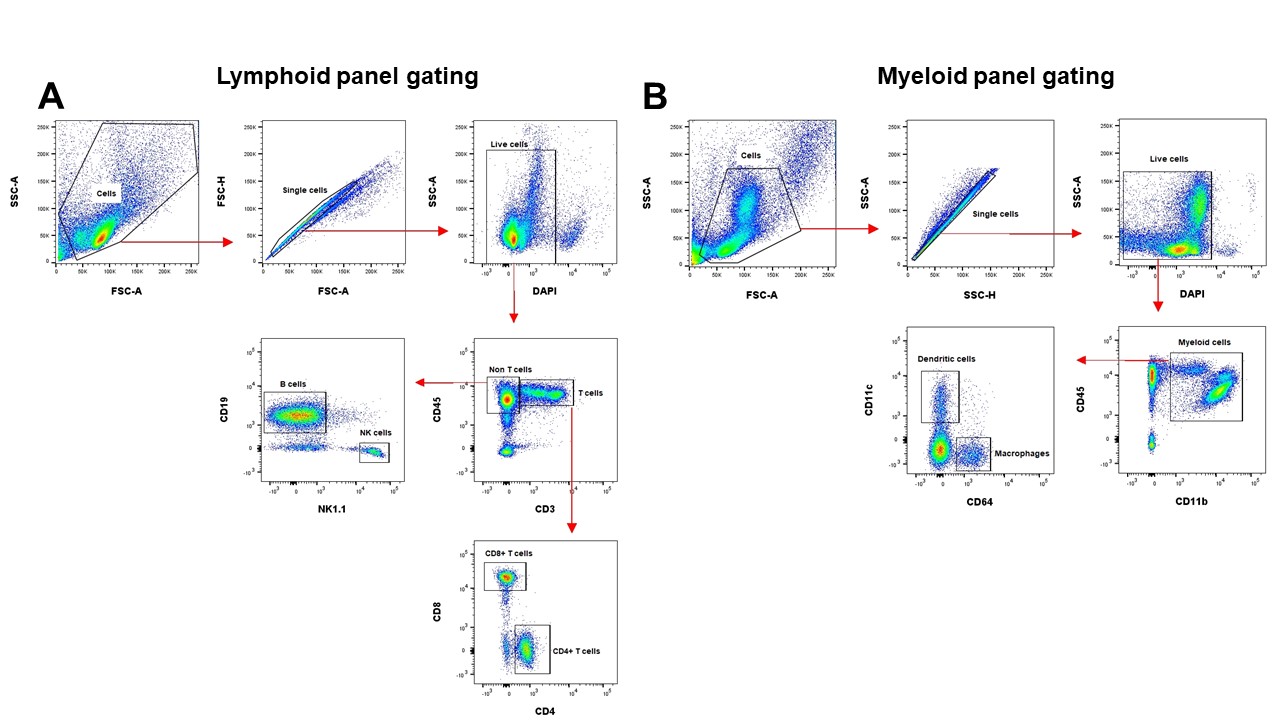
**

**Suppl. Fig. 1. Gating strategy for flow cytometry of the blood and spleen.** (A) Gating for lymphoid cell population. (B) Gating for myeloid cell population.


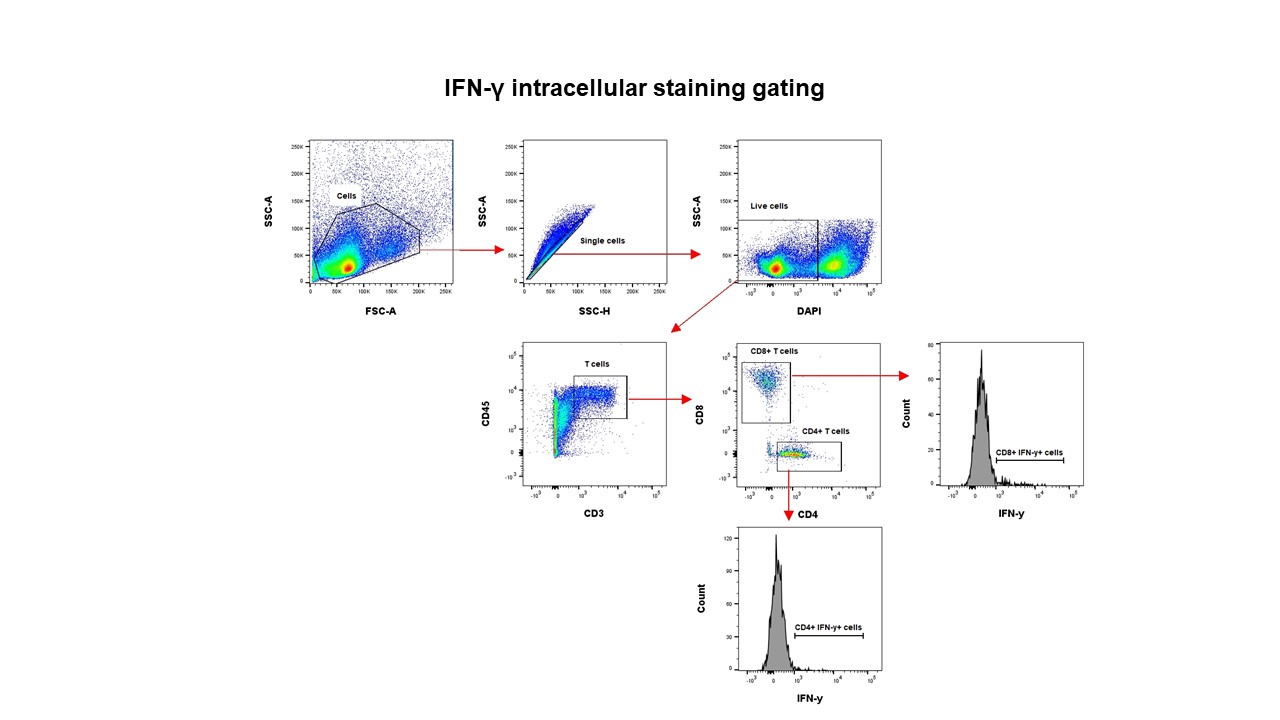


**Suppl. Fig. 2. Gating strategy for flow cytometry of splenocytes.** Gating for IFN-γ^+^ cells after intracellular staining.


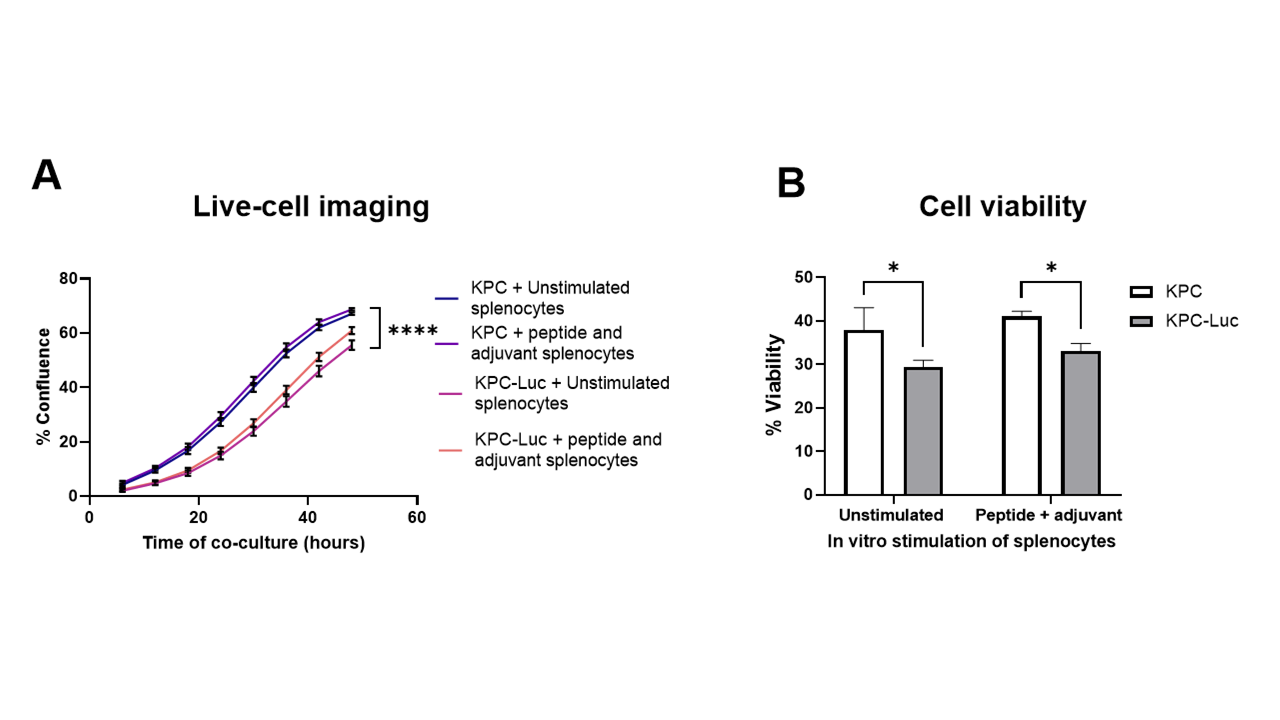
**Suppl. Fig. 3. Cytotoxicity induced by splenocytes from KPC-Luc tumor-bearing against KPC-Luc cells.** Splenocytes obtained from KPC-Luc mice, either unstimulated or incubated for 24 hours with both Luc peptide (LMYRFEEEL, 5 µg/ml) and the adjuvant R848 (2 µg/ml), were added to either KPC or KPC-Luc cells in a ratio of 1:20 per well (Effector: Target). (A) shows the percentage of confluence of KPC/KPC-Luc cells analyzed over 48 hours of incubation with splenocytes using a live-cell imaging system. (B) Cell viability at 48 hours of co-culture was measured by CellTiter-Glo assay. Two-way ANOVA. *p <0.05, ****p <0.001, n = 3.
